# Using a bodily illusion to examine the motivational basis of interoceptive hunger cues

**DOI:** 10.1007/s00426-025-02174-5

**Published:** 2025-09-08

**Authors:** Richard J. Stevenson, Daiana Martin-Rivera, Supreet Saluja, Heather M. Francis

**Affiliations:** School of Psychology, Macquarie University, Sydney, NSW2109 Australia

**Keywords:** Interoception, Hunger, Desire, Illusion, Stomach rumble

## Abstract

Internal bodily sensations such as an empty rumbling stomach can lead to enhanced desire for food - hunger. As an empty rumbling stomach is caused by digestive physiology, it is often presumed that such physiological processes also cause hunger. However, psychological processes could equally generate hunger (e.g., based on what the physiological signal has come to connote), and here we outline a method to test this idea. Participants were exposed to a stomach rumbling sound and control conditions (machine sound and silence) while they viewed pictures of food and everyday objects. For each picture, participants evaluated their desire to consume or own it, and judged, if there was a sound, where they felt it came from. Over half the participants experienced an illusion in which they mis-localised the stomach rumble sound to their own body. These participants also judged the pictures of food to be more desirable than comparable food pictures in the control conditions. This effect was obtained irrespective of when they had last eaten. Relative to control conditions, the stomach rumble sound had little impact on desire for pictures of everyday objects. These findings suggest it is possible to generate an illusory interoceptive state independent of physiology, which can then enhance desire to eat. This implies that purely psychological processes can generate bodily-cued desires.

**Supplementary Information:**

The online version contains supplementary material available at 10.1007/s00426-025-02174-5.

## Introduction

Interoception concerns the perception and processing of internal bodily states (Craig, 2002, 2003, 2009; Critchley et al., 2004; Harshaw, 2015; Wiens, 2005). Certain interoceptive states, such as an empty rumbling stomach or a dry mouth, can result in desire, in these cases for food and water respectively (e.g., Stevenson et al., 2015). An empty rumbling stomach and a dry mouth both result from physiological changes to the body that accompany the digestion of food and dehydration (e.g., Deloose et al., 2012; Rolls & Rolls, 1982). It would seem plausible that these same bodily physiological changes could also directly cause the accompanying psychological state of desire. There are two main reasons for this presumption. First, there are well characterised physiological pathways that lead from bodily change (e.g., empty stomach to ghrelin release; e.g., Kojima & Kangawa, 2005) to alterations in motivationally relevant brain systems (e.g., ghrelin stimulating hypothalamic structures; e.g., Wren et al., 2001). Second, there is the seemingly logical chain of events commencing with a physiological change to the body, with this always preceding the emergence of desire. However, this may not be a complete picture, because there is preliminary evidence that bodily-related desires might emerge from purely psychological processes (e.g., Stevenson et al., 2024). In this manuscript we outline a method to test this idea, and present evidence suggesting that psychological events may play an important role in the pathway between bodily physiological change and the emergence of desire.

The idea that psychological processes may play an important role in generating bodily desires has a long history (e.g., Changizi et al., 2002; Harshaw, 2008; Hebb, 1949; Richter, 1927). A key notion is that the meaning of certain bodily sensations may be learned. There is some evidence from animal and human studies that supports this view. In animals, rat pups do not respond to food and water deprivation with food and water seeking, until the deprivation state has been appropriately sated (Changizi et al., 2002). Here the animal seems to learn an association between the internal bodily state characterising deprivation, and the external agent that provides relief/reward (e.g., water, food). This would imply that the bodily sensations that occur during food and water deprivation are not intrinsically meaningful but that their meaning is acquired.

In humans, two lines of evidence suggest a similar conclusion. First, adults exhibit considerable variability in the type of bodily cues that they understand to mean hunger and thirst (e.g., Harris & Wardle, 1987; Monello & Mayer, 1967; Stevenson et al., 2024a). This variability is not random, as adult students and their parents are far more alike in how they manifest bodily thirst and hunger, than are adult students and randomly assigned adults of their parent’s age (e.g., Stevenson et al., 2023, 2024a). While this could reflect a genetically based similarity, this seems unlikely, as parents’ beliefs about the causes of hunger and thirst influence not only the bodily cues they use, but also the ones their children use as well (Stevenson et al., 2023, 2024a). This would suggest that parents somehow direct their child’s attention towards bodily cues they think are salient, suggesting a learning process. Second, studies of parents suggest that they do indeed notice external manifestations of their child’s internal bodily state, and that they also act upon this information (e.g., suggesting an appropriate course of action; Stevenson et al., 2024b). This suggests one route by which parents could assist children in learning what their interoceptive states mean.

If the meaning of interoceptive states are learned, then this offers a potential route for them to generate desire via psychological processes. For bodily sensations (i.e., interoceptive cues) that come to mean hunger, this acquisition of meaning is likely to be accompanied by reward. When someone experiences an empty rumbling stomach, if they eat shortly after, food will likely be rewarding. This allows for learning to take place between the interoceptive cue (empty rumbling stomach) and its consequences (food will be rewarding now). This form of learning should have two properties (Stevenson, 2024; Stevenson & Boutelle, 2024). The first concerns its specificity. When, for example, a person encounters a McDonalds or Coca-Cola logo, these external food cues are associated with a fairly specific class of rewards – burger and fries, and a sweet, cold, carbonated drink, respectively. In contrast, an empty rumbling stomach will have preceded many different types of food. Consequently, the association is less specific, being more for food in general. The second concerns consequences. An empty rumbling stomach is *never* encountered when sated with a full stomach. In contrast, external cues, like the logos for McDonalds or Coca-Cola, are encountered both when food or fluid deprived, and when sated. If consumption occurs when sated, the person learns that when the stomach feels full etc., food (or drink) is less rewarding. In this case the internal bodily state can come to modulate responses to these external cues (e.g., Davidson et al., 2019; Davidson & Stevenson, 2022). Such modulation should not occur for internal bodily cues as an empty stomach cannot simultaneously exist with a full one, so there is little opportunity for modulation to emerge. A hypothetical consequence of this absence of modulation, is that if one could stimulate the psychological pathway that connects an internal bodily cue to desire, then this should stimulate desire *irrespective* of actual bodily state.

A final issue is how an association between a bodily cue and its rewarding consequences results in desire. A first possibility is that an excitatory association is formed to the interoceptive cue (Stevenson, 2024; Stevenson et al., 2024a, b, c). Thus, when the interoceptive cue is encountered, this results in affectively positive food-related mental excitation. If an external food cue is then encountered (e.g., a McDonalds logo), the positive affect associated with this external cue is augmented by the concurrent mental excitation generated by the interoceptive cue. Thus, the external food cue would appear far more desirable, than it otherwise would. A second possibility can be derived from Schacter and Singer’s (1962) two-factor model of emotion, where cognitive appraisal of undifferentiated arousal or affect dictates the resulting emotional state. In this type of account, undifferentiated affect or arousal resulting from experiencing the interoceptive cue might then influence any subsequently affectively arousing event, *irrespective* of whether it is related or not (e.g., Baron & Bell, 1977; Bunce et al., 1993; Cantor et al., 1975; Laukkonen et al., 2022). Thus, an external food cue, encountered after the interoceptive cue, would be subject to transfer of excitation, and so again it would appear as more desirable. The primary difference between these two models, is that the first is more motivationally specific than the second. That is an interoceptive cue like an empty rumbling stomach might just increase desire for food (first model), or for food and other non-food related things as well (second model).

An ideal approach to study the issues addressed in this Introduction, would be to psychologically induce an interoceptive state independent of its normal physiological cause, and then examine the motivational impacts of this induction. This approach would allow us to address three questions. First, if psychological processes (irrespective of their form) are responsible for generating desire when a particular interoceptive cue occurs, then inducing the interoceptive cue psychologically should lead to enhanced desire. Second, enhanced desire should occur irrespective of whether the person has recently eaten or not (and relatedly whether they report being hungry or full), as modulation is not usual for interoceptive cues. Third, enhanced desire may be specific to food, or it may be more general including non-food objects - depending on which type of model - outlined above - is operative.

The key to addressing these three questions is whether it is possible to induce an interoceptive state psychologically. We have previously demonstrated that when the sound of an empty rumbling stomach is quietly played over a computer speaker, this sound is sometimes reported as being mis-localised to the participants own stomach, and when this occurs, participants report greater desire for food-related images (Stevenson et al., 2025). The occurrence of mis-localisation of the stomach rumble sound to one’s own stomach suggests the presence of a novel type of multisensory illusion. Like other multisensory illusions (e.g., Slater & Ehrsson, 2022; Tsakiris et al., 2011), not every participant seems to experience it, with its strength varying both across multiple trials within a person, and between individuals. In the current study, we build on this earlier work by presenting pictures of food and in addition, neutral everyday household objects, either accompanied by a stomach rumbling sound, a control machine sound - selected as it was both un-related to food and to the body - or no sound at all. After each presentation of a picture, we ask participants to evaluate their desire to consume/own the depicted object, and then where they felt the accompanying sound - if present – had come from. After completing this phase of the study, several other measures were collected, notably of participants evaluations of hunger/fullness/time since last meal, their ratings of the test sounds, especially what the stomach rumble sound meant (i.e., did it mean hunger), participants routine use of these type of interoceptive cues, and whether they felt any stomach rumble mimicry had occurred, as reported in our prior study (Stevenson et al., 2025).

Several effects then, are examined in the current study. First, we assessed whether the localisation ratings would provide evidence for the illusory mis-localisation of the stomach rumble sound to participant’s own body. As before this was tested when using food images (Stevenson et al., 2025) and now, here, non-food images too, with all responses compared to the control sound conditions. Second, we attempted to replicate our prior findings (Stevenson et al., 2025), by testing for a positive relationship between localisation ratings for the stomach rumble sound and desire to eat ratings for the food images. Third, and relatedly, we examined if desire to eat the food images would be greatest when contrasted to the other sound conditions, in those who experienced the stomach rumble illusion (i.e., mis-localisation of the sound to their own body). Fourth, we examined whether the stomach rumble sound would similarly affect desire for non-food objects. Fifth, we tested whether the hypothesised relationship between localisation ratings for the stomach rumble sound and desire to eat ratings of the food images, would occur independently of currently reported hunger, fullness, and time since last eating (i.e., state).

## Method

### Participants

We expected the predicted outcomes to fall in the medium effect size range (i.e., d ≈ 0.5 to 0.6), and so with power set at 0.8, we calculated that we would need between 90 and 120 participants to have an 80% chance of rejecting the null if H1 was true.

Participants were recruited from the first-year pool at Macquarie University and were asked to self-exclude if they had a history of eating disorders, were not of a healthy weight, or had a restrictive diet (e.g., vegan etc.). One hundred and twelve participants completed the study and reported complying with these inclusion criteria. During the study, participants engaged in two sound checks (detailed in the Procedure) in which they were presented with a pre-recorded spoken word via the computer speaker. They then had to type this word into the computer to ensure that their computer’s sound system was functioning effectively. Five participants failed this sound check suggesting they may not have heard the experimental sounds, leaving 107 cases for analysis. Of these 107 participants, 30 were male and 77 female, with a mean age of 19.6 years (SD = 3.5).

Participants were told that the study concerned how foods were perceived and judged. This was followed by a generic description of the tasks that they would be asked to undertake (e.g., you will be asked to listen to different sounds). All participants consented to take part, and the protocol was approved by the University IRB (approval number 520231576452157).

### Materials

Twenty-four neutral object colour images were obtained from the International Affective Picture Series (Lang et al., 2005), consisting of common household and everyday items (e.g., a side lamp, iron, hairdryer, car). Twenty-four colour food images, with items judged to be moderately palatable in a prior study (Stevenson et al., 2025), consisting of an equal number of sweet and savoury items, were obtained from internet searches.

Three sets of images were then assembled from this pool of 48 items. Each set, termed A, B and C, was composed of 8 unique food images (4 sweet and 4 savoury) and 8 unique neutral household images. Image sets (A, B, C) were then paired with the three sound conditions, resulting in six possible combinations (e.g., all A images with the stomach rumble sound, all B images with the machine sound, and all C images with no sound [silence]). Each combination was then used with equal frequency across participants, in this within-subject design, to ensure that any general tendency to prefer one set of images over another, did not affect the outcome.

The three sound conditions were composed of: (1) a stomach rumbling noise (6s duration, averaging 40 dBA); (2) a clacking typewriter/teleprinter machine noise (6s duration, averaging 50 dBA); and (3) silence. Note that the actual loudness of the sounds will vary somewhat between computers, even though we asked participants to set their volume to the same value.

### Procedure

The study was conducted online using Qualtrics, with the participant choosing when to complete it. No instructions were given regarding food or fluid intake prior to the study. This approach was adopted so that we could get accurate reports of when participants last ate that were not contaminated by demand characteristics.

Participants were instructed to use their computer speaker for the study and to set its volume to 40% of maximum, and to undertake the testing in a quiet space. The first sound check followed, in which a word was played out loud by the computer, with the participant having to type it in to confirm the sound system was working. They were then asked to report biographical details.

The main study task followed, starting with these general instructions: “You will now see a series of food and non-food images, some of which will be accompanied with a sound. Your job is to rate how much you would like to own or consume the item you just saw. You will also be asked to provide a rating of where you feel the sound you just heard came from. For example, if you heard people chatting, you might feel unsure where it came from – was it from the computer or from outside where you are?”. This was followed by 48 trials. Each trial involved the presentation of a picture on the screen for 8s, with this either accompanied by a sound (stomach rumble or machine noise; present for 6 of the 8s picture duration) or by no sound. Twenty-four of the pictures were of moderately palatable foods, with 8 paired with the stomach rumble, 8 with the machine sound and 8 with no sound. The remaining 24 pictures were of household and everyday items, with again 8 of these paired with the stomach rumble, 8 with the machine sound and 8 with no sound. Order of presentation of picture-sound/silence pairs was randomised for each participant.

After each picture-sound/silence presentation, participants completed two ratings in the form described below (i.e., the question/rating format was the same for all trials). First, they rated their desire to own or consume the depicted item (How much would you like to own or consume this now? – 7 point category scale, anchors Not at all [1], Very much [7]). Second, participants judged the perceived location of the sound – if there was one (Did you *feel* the sound came from the computer or from you or the environment? – 7 point category scale, anchors, From computer [1], Unsure/No sound [4], From me or environment [7]). After the second rating was finished, the next trial commenced, with this process repeated until all 48 trials had been undertaken.

A second sound check was then completed, with the computer saying out loud a different word to that used in the first sound check. The two test sounds - stomach rumble and machine - were then evaluated. After each sound played, participants judged: (i) how loud it was (1 to 7 scale, with anchors ‘Not at all’ to ‘Very’); (ii) how much they liked the sound (1 to 7 scale, with anchors [1] ‘Not at all’, [4] ‘Neither like nor dislike’, to [7] ‘Very much’); (iii) how arousing it was (1 to 7 scale, with anchors [1] ‘Not at all’ to [7] ‘Very’); (iv) what they thought the sound was; and (v) what this sound meant to them. Responses to these last two questions were open and were coded by the presence of the following labels for identification (stomach rumble; machine noise), and for meaning (hunger for stomach rumble; machine-related noise for machine sound).

Data on current state was then collected. Participants were asked how long ago they last ate (in minutes), how hungry they were now (7 point category scale, anchors [1] Not at all, [7] Very) and how full (7 point category scale, anchors [1] Not at all, [7] Very). Next, they were asked if they had ever experienced their stomach rumbling *after* hearing the stomach rumbling sound to assess if they had experienced any possible mimicry (i.e., noticing their own stomach rumble shortly after hearing the sound of a stomach rumbling). Responses were made on a five-point category scale – No-Never, Yes-Rarely, Yes-Sometimes, Yes-Most of the time, and Yes-Always. Participants then reported their height and body weight for the calculation of body mass index (BMI).

Finally, a modified version of the Monello and Mayer (1967) hunger questionnaire was presented. This consisted of four blocks of questions. Each block had the same question format: ‘When you are hungry, which of the following sensations do you experience?’. Bodily sensations were arranged into the following four blocks: Stomach (6 items) – with the first question focussing on emptiness and the second on rumbling – the two items used here; after which came blocks for Mood (10 items); Mouth/Throat (5 items); and General/Head (7 items). Participants rated the presence of each of these items when hungry, using a six-point category scale (0 = Not at all, 1 = Very weak, 2 = Weak, 3 = Moderate, 4 = Strong and 5 = Very strong) – the key modification from the original scale which used a yes/no response format.

### Analysis

Participants state data is reported first (i.e., hunger, fullness, and the time since they last ate) both to present its variability (i.e., range/SD) and inter-relatedness (i.e., correlations). The former was important as variability is necessary to test if state mediates the relationship between stomach rumble localisation ratings and food desire. Correlations were included to see if we could use a data reduction strategy (i.e., factor analysis) to generate a single variable to reflect state.

Ratings of the two test sounds – stomach rumble and machine – for loudness, liking, and arousal, were compared using paired-sample t-tests. The proportion correctly identifying each sound, and their meaning, are also reported.

Localisation ratings were the examined to see if illusory mis-localisation of the stomach rumble sound to participant’s own body occurred with the food images and for the non-food images, and how these ratings compared to the other sound conditions. Six mean localisation scores were computed for each participant, these being the averages for each picture type (food vs. household) by sound condition (stomach rumble vs. machine sound vs. silence). These ratings were examined using Bonferroni adjusted paired-sample t-tests, as our initial focus was whether the type of picture influenced localisation ratings for the stomach rumble sound, and then whether illusory localisation to the body was greater for the stomach rumble sound, relative to the machine sound, when tested for the food pictures, and for the neutral household pictures. This was followed by a descriptive analysis of the localisation rating response profiles for each of the picture and sound conditions, to illustrate the differences across them.

Participants desire ratings for each picture type (food vs. household) by sound condition (stomach rumble vs. machine sound vs. silence), were then examined using a two-way repeated measures ANOVA. This method was appropriate here as there were no specific contrasts of interest at the aggregate level (i.e., not taking account of differences in localisation ratings for the stomach rumble sound).

To test the relationship between localisation ratings for the stomach rumble sound and desire to eat ratings for the food images, we used a series of regression analyses. These analyses had three components. The first, was the inclusion on each regression analysis of certain predictor variables to establish if stomach rumble localisation alone was uniquely related to food desire judgments. These predictor variables were the characteristics of the stomach rumble sound (loudness, liking, arousal, identification, meaning; all of which might impact desire to eat/consume), and whether participants normally used the stomach rumble as a hunger cue (from the Monello and Mayer questionnaire), which might influence whether or not this cue was effective for them. In addition, and to test the impact of participant’s state, a factor derived measure of state was included in the regressions, alongside an interaction term of state by localisation score (note that removing the interaction term has no effect on any of the reported analyses).

The second component of the regression analyses was to take into account the impact of the other sound conditions (i.e., silence and machine). An initial regression analysis was undertaken, with the dependent variable being participants food image desire ratings obtained with the stomach rumble sounds. This was followed by a second regression analysis, which utilised stomach rumble food desire ratings but now minus those obtained on the silence condition, as the dependent variable. A third regression analysis was then undertaken, with the dependent variable being stomach rumble food desire ratings now minus those obtained on the machine condition. This series of analyses then, ensured that any relationship observed between food desire and stomach rumble localisation score, was unique to that condition.

The third component of the regression analysis examined whether there was a relationship between stomach rumble localisation, and desire for the neutral household objects. The same basic approach, as just described for the food images, was used here.

To more directly assess whether participants who mis-localised the stomach rumble sound to their own stomach judged the accompanying food images to be more desirable, we undertook two further sets of analyses. Here, desire to eat ratings for the stomach rumble and control sound conditions were contrasted between sub-groups of participants (i.e., those who localised the stomach rumble to their own stomach vs. those who did not) using independent and one-sample t-tests, with Bonferroni correction.

The final part of the analysis undertook a more formal test, to establish whether participants state, mediated the relationship between stomach rumble localisation score and food desire ratings, using the Sobel test. This final test used both the factor derived state score as a mediator, and the time since the last meal, to try and ensure there was no potential contamination of our measure of state by the stomach rumble manipulation (i.e., this might have affected hunger/fullness ratings, but would presumably have less impact on recalling when the participant last ate).

## Results

### State

Participants varied markedly in how hungry and full they were, and when they last ate. Responses on the hunger rating scale varied from 1 to 7, with a mean of 3.5 (SD = 1.7), and responses on the fullness rating scale also varied from 1 to 7, with a mean of 3.8 (SD = 1.6). On average, participants ate just over 2h before the study (M = 129min, SD = 182min), with this ranging from 0min to 24h before. These three variables were all significantly correlated (all r’s > ± 0.24; with a coefficient alpha = 0.67), and so we used factor analysis (data were factorable, with Kaiser-Meyer-Olkin index > 0.5 and a significant Bartlett’s test) to determine their structure. This revealed just one factor, and so we then generated a regression-derived factor score of state, for use in the later analyses.

### Sounds

The sound evaluation data are presented in Table 1. The machine sound was judged significantly louder than the stomach rumble (t(106) = 19.45, *p* < 0.001, d = 1.88), but they did not differ in liking (t = 1.48), with both sounds judged equally negative. The stomach rumble sound was judged as significantly more arousing than the machine sound (t(106) = 3.08, *p* = 0.003, d = 0.30). Most participants identified both sounds, along with their meaning, and these proportions did not significantly differ between sounds.

**Table 1:** Sound evaluation data

Measure	Stomach rumble sound	Machine sound
Loudness, mean (SD)	3.8 (1.2)	5.9 (1.2)
Liking, mean (SD)	2.8 (1.3)	2.5 (1.3)
Arousal, mean (SD)	2.2 (1.5)	1.7 (1.3)
Identification, % reporting (correct response)	85.6% (Stomach rumble)	87.4% (Machine sound)
Meaning, % reporting (correct response)	77.1% (Hunger for food)	80.9% (Machine’s action)

### The source of the sound - localisation judgments

Participant reports of where the sound came from – if there was a sound – are presented in Table 2. For the stomach rumble, mean localisation scores were significantly higher (i.e., more to self) when presented with food pictures (M = 3.2, SD = 1.8), than with neutral household pictures (M = 2.7, SD = 1.7)=; t(106) = 4.45, *p* < 0.001, d = 0.43, critical alpha = 0.017). When the stomach rumble sound was presented with food pictures, there was significantly greater mis-localisation to self (M = 3.2, SD = 1.8) than when the machine sound was presented with the food pictures (M = 1.6, SD = 1.1; t(106) = 9.45, *p* < 0.001, d = 0.91, critical alpha = 0.017). Similarly, when the stomach rumble sound was presented with neutral household pictures (M = 2.7, SD = 1.7), there was significantly more mis-localisation of the sound to self than when the machine sound was presented with the neutral household pictures (M = 1.8, SD = 1.2; t(106) = 6.30, *p* < 0.001, d = 0.61, critical alpha = 0.017). For the silence condition, ratings were around 4, which was the appropriate response when a sound could not be detected.

**Table 2:** Reports of sound location by sound type and picture type

Measure						
Stomach rumble sound			Machine sound		Silence	
Food		Household	Food	Household	Food	Household
Mean localisation score (SD)						
3.2 (1.8)		2.7 (1.7)	1.6 (1.1)	1.8 (1.2)	3.8 (0.5)	3.6 (0.6)
Percent of participants with *at least one* score of 5+ (i.e., mis-localisation to self/environment)						
61.7%		45.8%	18.7%	22.4%	20.6%	11.2%
Percent of all localisation scores that are 1, 2 or 3 (i.e., source is likely the computer)						
58.3%		67.8%	90.6%	87.8%	12.9%	16.5%
Percent of all localisation scores that are 4 (i.e., unsure of source/no sound)						
6.2%		6.5%	2.5%	2.7%	83.0%	81.5%
Percent of all localisation scores that are 5, 6 or 7 (i.e., source is likely self/environment)						
35.5%		25.7%	6.9%	9.5%	4.1%	2.0%

Sixty-two percent of the sample experienced at least one episode of mis-localisation of the stomach rumble sound to self (i.e., giving one or more responses of 5, 6 or 7) when presented with food images, and 46% did so when presented with household images. In contrast, only 19%, 22%, 21% and 11% reported similar scores of 5, 6 or 7, in the other conditions – see Table 2.

Table 2 also illustrates the percentage of responses falling in different parts of the localisation scale. The resultant pattern of results is quite distinct for each sound condition. For the stomach rumble, between a third (35.5% - food images) and a quarter (25.7% - neutral household images) of all responses indicate some degree of mis-localisation of the stomach rumble sound to self. For the machine sound, this was overwhelmingly judged as coming from the computer, when presented either with food (6.9%) or neutral household pictures (9.5%). For the silence condition, the modal response was 4 (i.e., 83.0% - food images; 81.5% - neutral household images). A response of 4 indicates on the rating scale that the participant could not hear a sound.

Participants were asked at the end of the study if they had experienced their stomach rumbling *after* hearing the stomach rumbling sound, namely if they had possibly experienced mimicry. Just over half reported that this had happened to them, with 18.8% responding that it had happened rarely, 22.3% sometimes, and 6.3% most of the time. Thus, over half the participants reported experiencing their stomach rumbling after hearing the sound on the computer.

### Desire to consume/own judgments

The desire ratings (see Table 3) were analysed using a two-way repeated measures ANOVA. The analysis revealed a main effect of Picture type (F(1,106) = 519.53, *p* < 0.001, partial eta-squared = 0.83) with higher desire ratings for the food compared to the neutral household images (see Table 3), and a main effect of Sound condition (F(2,212) = 5.67, *p* = 0.004, partial eta-squared = 0.051). For the Sound condition, Bonferroni adjusted contrasts, indicated that images viewed in the silence condition (M = 3.1, SD = 1.1) were judged as more desirable than those in the stomach rumble (M = 3.0, SD = 1.0; *p* < 0.013) and machine conditions (M = 3.0, SD = 1.1; *p* < 0.013) – with the impact of these two sounds on desire judgments not significantly differing. There were no other effects.

**Table 3:** Evaluations of the pictures, by sound condition

Measure						
Stomach rumble sound			Machine sound		Silence	
Food		Household	Food	Household	Food	Household
Mean desire (SD)						
	4.1 (1.1)	1.8 (1.0)	4.1 (1.1)	1.9 (1.0)	4.4 (1.2)	2.0 (1.0)

In sum, both food and neutral household pictures tended to be judged as less desirable when presented with either stomach rumble or machine sounds.

### Food desire in the stomach rumble condition

**Fig. 1:**
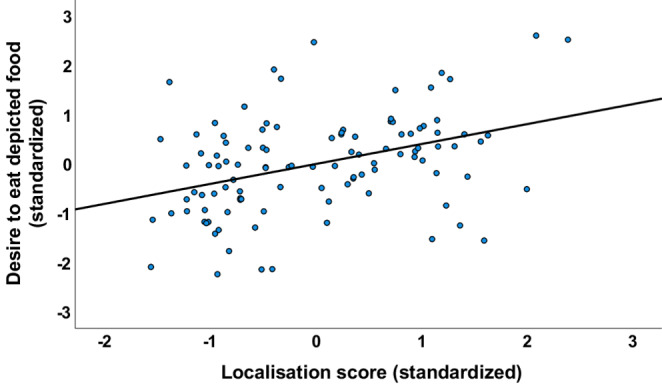
Partial regression plot of the relationship between desire to eat the depicted food and localisation score for the rumbling stomach sound (more positive score = greater mis-localisation to self)

We explored predictors of the food desire ratings in the stomach rumble condition using multiple regression with simultaneous entry (see Table 4 for predictors). The overall model was significant (F(9,97) = 5.44, *p* < 0.001) and explained 27.4% of the variability in food desire ratings (adjusted R^2^) for the stomach rumble sound condition. All the predictor variables - see Table 4 - were positively associated with desire to eat ratings, with most significantly so. Only two variables were uniquely predictive in the overall model. These were localisation score, with greater mis-localisation to self, associated with greater desire to eat ratings (see Fig. 1), and the aggregate measure of state (i.e., hunger) with greater hunger linked to greater desire to consume the depicted foods. There was no interaction between these two variables.

**Table 4:** Predictors of desire to eat for the food images in the stomach rumble sound condition

Variable	*r*_0_ with DV	B (SE)	beta	Sr^2^%	*p* for Sr^2^
Localisation score	0.45*	0.40 (0.10)	0.36	11.8	<0.001
Rumble loudness	0.16*	0.10 (0.09)	0.09	0.7	0.31
Rumble like	0.19*	0.06 (0.08)	0.08	0.4	0.44
Rumble arousal	0.13	0.02 (0.07)	0.03	0.0	0.73
Rumble identified	0.26*	0.61 (0.33)	0.19	2.4	0.065
Rumble meaning	0.22*	0.06 (0.28)	0.02	0.0	0.82
Use of this cue†	0.22*	0.10 (0.08)	0.11	1.0	0.22
State	0.32*	0.22 (0.10)	0.19	3.2	0.033
State X Localisation score	0.09	0.06 (0.10)	0.05	0.2	0.62

*Significant zero order correlation with the dependent variable

†Mean of the two relevant questions from the Monello and Mayer survey

### Food desire contrasted between the stomach rumble condition and the silent condition

Using multiple regression, we examined the predictors of the *difference* between the food picture desire judgments made when the stomach rumble was present contrasted with (i.e., subtracted from) when the food pictures were judged in silence (see Table 5). The overall model was significant (F(9,97) = 2.35, *p* = 0.019), accounting for 10.3% of the variance of the difference in desire ratings between these two conditions. Four of the predictors had significant positive zero order correlations with the difference score, and of these, two were significant unique predictors in the regression model. Greater mis-localisation of the stomach rumble to self was associated with greater desire to consume the food images in the stomach rumble condition (relative to the silence condition), as was reporting that the stomach rumble meant hunger.

**Table 5:** Predictors of desire to eat for the food images in the stomach rumble sound condition contrasted with - minus - desire to eat for the food images in the silent condition

Variable	*r*_0_ with DV	B (SE)	beta	Sr^2^%	*p* for Sr^2^
Localisation score	0.22*	0.24 (0.11)	0.20	3.8	0.036
Rumble loudness	0.04	−0.04 (0.11)	−0.04	0.1	0.71
Rumble like	0.22*	0.18 (0.09)	0.20	3.1	0.057
Rumble arousal	0.02	−0.07 (0.08)	−0.10	0.7	0.36
Rumble identified	0.23*	0.15 (0.37)	0.04	0.1	0.70
Rumble meaning	0.31*	0.71 (0.32)	0.26	4.2	0.027
Use of this cue†	0.10	0.06 (0.09)	0.07	0.4	0.50
State	0.07	−0.07 (0.11)	−0.06	0.3	0.54
State X Localisation score	0.01	0.01 (0.12)	0.01	0.0	0.91

*Significant [*p* < 0.05] zero order correlation with the dependent variable

†Mean of the two relevant questions from the Monello and Mayer survey

We then tested just those participants who reported that the stomach rumble sound meant hunger. We determined if the difference in their desire to eat ratings between the stomach rumble sound contrasted with the silent condition, was positive in those demonstrating mis-localisation of the stomach rumble to self (i.e., scoring > 4.1 [after rounding]). Fifty participants had a mean localisation score < 4.0, and the mean difference in food desire ratings between the stomach rumble and silence condition was − 0.33 (SD = 0.9). However, for the 33 participants with a mean localisation score > 4.1, the mean difference in food desire ratings between the stomach rumble and silence condition was 0.43 (SD = 1.0). Not only did the latter mean (0.43) significantly exceed the former (−0.33; t(81) = 3.51, *p* = 0.001, d = 0.78; critical alpha = 0.025), a one-sample t-test (mu = 0) indicated that participants with a localisation score > 4.1 also desired the food images more in the stomach rumble condition than in the silent condition (t(32) = 2.37, *p* = 0.024, d = 0.41; critical alpha = 0.025). Thus, when participants know what the stomach rumble means, and mis-localise this sound to themselves, food desire is greater in this condition than in the silent condition.

### Food desire contrasted between the stomach rumble condition and the machine condition

Using regression, we explored the predictors of the difference between the food picture desire judgments made when the stomach rumble was present contrasted with (i.e., subtracted from) when the food pictures were judged with the machine sound present (see Table 6). The overall model was significant (F(9,97) = 2.31, *p* = 0.021), and accounted for 10.0% of the variance in the difference in desire ratings between these two conditions. Four of the predictors had significant positive zero order correlations with this difference score, and of these two were significant unique predictors in the regression model. Greater mis-localisation of the stomach rumble to self was associated with greater desire to consume the food images in the stomach rumble condition, as was reporting that it meant hunger.

**Table 6:** Predictors of desire to eat for the food images in the stomach rumble sound condition contrasted with - minus - desire to eat for the food images in the machine condition

Variable	*r*_0_ with DV	B (SE)	beta	Sr^2^%	*p* for Sr^2^
Localisation score	0.24*	0.14 (0.05)	0.25	5.9	0.010
Loudness difference†	0.02	0.06 (0.09)	0.07	0.3	0.50
Liking difference†	−0.18*	−0.09 (0.06)	−0.18	2.4	0.094
Arousal difference†	−0.04	0.06 (0.01)	0.15	0.0	0.99
Rumble identified	0.20*	0.07 (0.34)	0.02	0.0	0.85
Rumble meaning	0.26*	0.57 (0.29)	0.24	3.4	0.048
Use of this cue††	0.12	0.06 (0.08)	0.08	0.6	0.41
State	0.01	−0.14 (0.10)	−0.14	1.6	0.17
State X Localisation score	0.06	0.10 (0.10)	0.09	0.7	0.36

*Significant [*p* < 0.05] zero order correlation with the dependent variable

†Difference between rumble and machine evaluations

††Mean of the two relevant questions from the Monello and Mayer survey

We then tested just the participants who reported that the stomach rumble sound meant hunger. We tested if the difference in desire to eat ratings between the stomach rumble sound contrasted with the machine condition, was positive in those demonstrating mis-localisation of the stomach rumble to self (i.e., scoring > 4.1 [after rounding]). Fifty participants had a mean localisation score < 4.0, and their mean difference in food desire ratings between stomach rumble and silence condition was − 0.13 (SD = 0.9). Thirty-three participants had a mean localisation score > 4.1, and their mean difference in food desire ratings between the stomach rumble and silence condition was 0.59 (SD = 1.0). Not only did the latter mean (0.59) significantly exceed the former (−0.13; t(81) = 3.42, *p* = 0.001, d = 0.77, critical alpha = 0.025), a one-sample t-test (mu = 0) indicated that participants with a localisation score > 4.1 also desired the food images more in the stomach rumble condition than in the machine sound condition (t(32) = 3.40, *p* = 0.002, d = 0.59, critical alpha = 0.025). Thus, when participants know what the stomach rumble means, and mis-localise this sound to themselves, food desire is greater in this condition than in the machine sound condition.

### Desire for the household objects in the stomach rumble condition

**Fig. 2:**
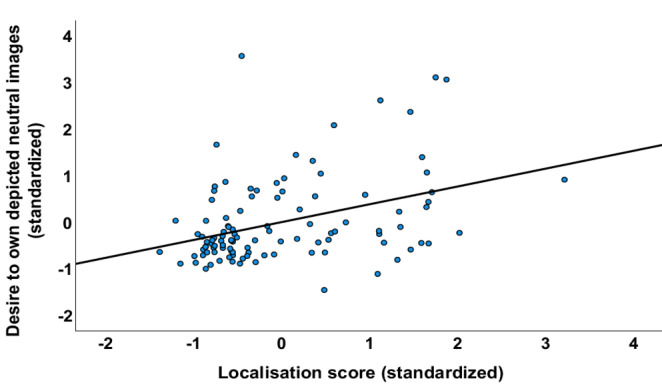
Partial regression plot of the relationship between desire to own the depicted neutral household items and localisation score for the rumbling stomach sound (more positive score = greater mis-localisation to self)

Using multiple regression, with desire for the household objects in the stomach rumble condition as the DV, revealed a significant overall model (F(9,97) = 3.30, *p* < 0.001), which explained 27.4% of the variability (adjusted R2) in desire. Three predictor variables - see Table 7 - were significantly associated with desire for the household objects, but only one variable was uniquely predictive in the overall model. This was localisation score, with greater mis-localisation of the stomach rumble to self, associated with greater desire for the household objects (see Fig. 2).

**Table 7:** Predictors of desire for the neutral household images in the stomach rumble sound condition

Variable	*r*_0_ with DV	B (SE)	beta	Sr^2^%	*p* for Sr^2^
Localisation score	0.43*	0.38 (0.09)	0.39	13.0	<0.001
Rumble loudness	−0.01	−0.01 (0.09)	−0.01	0.0	0.91
Rumble like	0.06	−0.02 (0.08)	−0.02	0.0	0.84
Rumble arousal	0.16*	0.11 (0.06)	0.18	2.4	0.084
Rumble identified	−0.10	0.16 (0.31)	0.06	0.2	0.62
Rumble meaning	−0.15	−0.41 (0.22)	−0.17	1.9	0.12
Use of this cue†	−0.06	−0.04 (0.09)	−0.04	0.2	0.67
State	0.12	0.06 (0.09)	0.06	0.4	0.51
State X Localisation score	0.16*	0.07 (0.10)	0.07	0.5	0.46

*Significant [*p* < 0.05] zero order correlation with the dependent variable

†Mean of the two relevant questions from the Monello and Mayer survey

### Desire for the household objects contrasted between the stomach rumble and silent conditions

Using the same predictor variables as in the preceding analysis, and with the difference in desire between the stomach rumble and silent conditions as the dependent variable, we conducted a further regression analysis. In this case the overall model was not significant (*p* = 0.16).

### Desire for the household objects contrasted between the stomach rumble and machine conditions

Using the same predictor variables as in the comparable analysis for food pictures, and with the difference in desire between the stomach rumble and machine conditions as the dependent variable, we conducted a final regression analysis. The overall model was not significant (*p* = 0.48).

### Mediation of the stomach rumble localisation-food desire relationship by state

To examine more formally if state was indirectly mediating the relationship between stomach rumble localisation score and desire ratings for food, we conducted a Sobel test. The Sobel test was not significant. Because our aggregate measure of state contained ratings of hunger, which might reasonably be influenced by the preceding manipulations – as with fullness ratings - we repeated the mediation analysis using just the reported time since last eating. Again, the Sobel test was not significant. These findings suggest that state – measured either as an aggregate of hunger, fullness and time since the last meal, or just the time since the last meal – does not significantly influence the magnitude of the relationship between stomach rumble localisation and food desire.

## Discussion

Here, we report an examination of whether those who know that the stomach rumble sound means hunger, and who experience mis-localisation of this sound to self, would: (1) judge food, and non-food household objects, as more desirable than when presented with a control sound or silence; and (2) would show the latter effect irrespective of when they last ate (and relatedly of their current state of hunger and fullness). Overall, we found that 62% of participants reported one or more occasion on which they judged the stomach rumble sound to have arisen from their own body. This suggests that most participants are susceptible to this illusion, even under what are probably non-ideal on-line conditions (e.g., variation in computer sound experience, adherence to the procedure etc.). In those experiencing mis-localisation, and who also understood the sound to mean hunger, ratings of desire to eat were higher when the stomach rumble condition was contrasted to both the control machine sound condition, and to the silence condition – replicating our previous finding (Stevenson et al., 2025). These effects were not present in participants who did not report mis-localisation and who thought the sound meant something else – often that being something negative (i.e., sickness). There was only limited evidence favouring the idea that the stomach rumble sound could facilitate desire for neutral household objects (i.e., regression results in Table 7), suggesting that the effect may be specific to food. Finally, using both an aggregate measure of state (hunger) and time since the last meal as a sole variable, we found that the effects of mis-localisation on food desire were independent of these variables (i.e., food desire could be augmented irrespective of current hunger/when the person last ate).

Before examining the implications of these findings, it is important to consider their limitations. As noted above, data were collected on-line, with most participants completing the study at home on their own computer. Consequently, there is likely variation in both the loudness and quality of the sounds, with this variability presumably working against demonstrating an effect. In addition, we did not experimentally manipulate state (i.e., feed participants), and so our findings in this respect are dependent on the accuracy of reports of hunger, fullness, and time since their last meal. While we avoided specifying any period of food deprivation prior to testing, to minimise demand characteristics, there still would have been variation in what was eaten at that last prior meal (e.g., a big versus a small recent meal). Notwithstanding, the aggregate hunger measure was still correlated with food desire judgments, suggesting sufficient sensitivity to detect a relationship. A further consideration is that our key findings are correlational. While we acknowledge these limitations, we note that the desire-related food findings replicate those of our prior study (Stevenson et al., 2025), our findings here are internally consistent, and they agree with general theoretical predictions made before the study was undertaken (Stevenson, 2024; Stevenson et al., 2024a, b, c).

The key manipulation here was presenting participants with the sound of a human stomach rumble at around 40 dBA (i.e., as loud as a quiet office or a refrigerator hum) – but noting loudness would have varied across participant computers. When participants heard this sound on the 8 trials with food pictures, 62% reported at least one occasion when it appeared to come from their stomach rather than the computer’s speaker. A smaller 46% did so on the 8 trials this sound appeared with neutral household objects. Reports of mis-localisation were significantly higher for the stomach rumble sound than they were for the control machine sound, suggesting that this was probably a specific property of the stomach rumble sound rather than a general effect of any sound. While participants clearly knew that the source of the sound was the computer, in just the same way than an audience knows that it is the ventriloquist, not the dummy that is the source of the dummy’s voice (e.g., Bertelson et al., 2000), the illusion occurs regardless. We suggest the effect here is similar, in that an ambiguous sound that *could potentially* come from self or computer, can be mis-localised to self, in the same way that the ventriloquist’s voice is mis-localised to the dummy. Presumably under conditions that maximise the ambiguity of the stomach rumble sound, one would expect a greater proportion of participants to experience illusory mis-localisation.

We also noted another effect, namely that just over half the participants reported experiencing their stomach rumble *after* hearing the computer-generated sound. While this ‘after’ effect was significantly correlated with instances of mis-localisation (*r* = 0.48), the absence of a perfect correlation implies participants who experienced this ‘after’ effect independent of instances of mis-localisation. One possibility is that this ‘after’ effect is a consequence of attending to this type of sound, resulting in increased vigilance for its occurrence. This could certainly account for some cases. However, given the extensive literature documenting mimicry for both volitional (e.g., posture) and non-volitional (e.g., pupil diameter) systems (e.g., Palagi et al., 2020), as well as plenty of evidence showing that mimicry can occur for yawning (Norscia & Palagi, 2011), and that it is possible to vicariously experience itch and pain (Grice-Jackson et al., 2017; Schut et al., 2016) – this would suggest that mimicry for stomach rumbling may also be possible.

A key rationale for this study was to test the idea that psychological processes may be important in moving from the occurrence of an interceptive cue to the emergence of desire (Stevenson et al., 2024a, b, c). Previously, it has not been possible to test psychological processes without concurrently changing physiological state (an observation also made by other investigators; e.g., Tsakiris, 2017). This creates an inherent explanatory confound, as a change in physiological state always precedes the psychological effect, making it hard to rule out a physiological cause. In the current study, participants who knew what the stomach rumble sound meant, and who experienced illusory mis-localisation, judged pictures of food as more desirable than comparable pictures presented with silence or with a machine sound. As physiological state remained unchanged, and was not manipulated here, the inference is that experiencing an illusory stomach rumble led to an increase in food desire via a psychological process. This suggests – tentatively of course given the preliminary nature of our findings - that purely psychological pathways can enact increases in desire following an interoceptive cue.

The study provides the first evidence for how this psychological pathway might unfold. In the introduction we outlined two models, one dependent on an associative process, in which the interoceptive cue brings to mind rewarding food-related mental excitation. This excitation could then augment affect associated with any external food-related cue – like pictures or smells - so that these becomes more desirable. The other model assumed a more generic form of representation produced by the interoceptive cue, one potentially capable of augmenting both external food and non-food related cues. The data from this study favours the first possibility, as while we obtained evidence of enhanced food desire in those who mis-localised the stomach rumbling sound and understood its meaning, there was no consistent evidence for this with neutral household images. While there was an association between the degree of stomach rumble mis-localisation and enhanced desire for the neutral household images which is suggestive, this effect was not sustained when contrasts were made to the control machine sound condition, and the silence condition. Of course, the choice of images here could have worked against a general enhancement effect. Perhaps if pictures of more positively valanced non-food items had been used (e.g., money, and money-related objects), then an effect might have been observed.

A second key issue was also examined, namely the state of the participant (i.e., hunger/fullness/time since last meal) at the time of testing. We suggested in the Introduction that because interoceptive hunger cues are not normally modulated (i.e., you cannot normally have an empty and full stomach simultaneously), they may be able to enhance desire irrespective of whether the participant has recently eaten. First, using an aggregate measure of state, based on ratings of hunger, fullness and time since last eating, we could find no evidence of modulation. Second, as an additional test, we also included an interaction effect between the mis-localisation score and the aggregate hunger measure in the regression analyses, to see if desire ratings might be especially high when mis-localisation combined with state (i.e., hunger). There was no evidence for this either. Third, we undertook a direct test of mediation using a Sobel test, both with the aggregate state measure, and using just the time since last eating, to minimise any possible contamination of hunger/fullness measures by the main experimental manipulations. Both Sobel tests were non-significant, suggesting no evidence of mediation. A more robust experimental test is now required, to see if feeding participants influences the effect of mis-localisation on desire, when tested before and after a meal. Finally, while this finding of independence draws upon a null result, we noted earlier that the aggregate measure of hunger substantially correlated with food desire ratings (see Table 4), suggesting our procedure was sensitive enough to detect this effect.

In conclusion, it has long been suspected that psychological processes, especially those connected with learning and memory, may contribute to the motivational basis of interoceptive hunger cues (e.g., Changizi et al., 2002; Harshaw, 2008; Hebb, 1949; Richter, 1927). Neuropsychological data also suggest this. Patients with semantic dementia are sometimes unable to understand the meaning of their interoceptive states (Gan et al., 2016), while damage to the medial temporal lobes – critical for learning and memory – is linked to an inability to experience or utilise interoceptive hunger cues (e.g., Hebben et al., 1985). These types of findings are problematic for the idea that physiological processes alone cause the experience of hunger – desire for food. Rather the findings here suggest that psychological processes involving learning and memory, may be important causes of interoceptive hunger, and possibly of other desires linked to interoceptive states.

## Supplementary Information

Below is the link to the electronic supplementary material.

Supplementary Material 1

## Data Availability

Data is available as a supplementary file.
